# Multiple Hsp70 Isoforms in the Eukaryotic Cytosol: Mere Redundancy or Functional Specificity?

**DOI:** 10.2174/138920208785133280

**Published:** 2008-08

**Authors:** Mehdi Kabani, Céline N Martineau

**Affiliations:** Laboratoire d’Enzymologie et Biochimie Structurales (LEBS), CNRS, Bât. 34, Avenue de la Terrasse, 91198 Gif-sur-Yvette, France

**Keywords:** Hsp70, Ssa1, chaperone network, functional specificity.

## Abstract

Hsp70 molecular chaperones play a variety of functions in every organism, cell type and organelle, and their activities have been implicated in a number of human pathologies, ranging from cancer to neurodegenerative diseases. The functions, regulations and structure of Hsp70s were intensively studied for about three decades, yet much still remains to be learned about these essential folding enzymes. Genome sequencing efforts revealed that most genomes contain multiple members of the Hsp70 family, some of which co-exist in the same cellular compartment. For example, the human cytosol and nucleus contain six highly homologous Hsp70 proteins while the yeast *Saccharomyces cerevisiae *contains four canonical Hsp70s and three fungal-specific ribosome-associated and specialized Hsp70s. The reasons and significance of the requirement for multiple Hsp70s is still a subject of debate. It has been postulated for a long time that these Hsp70 isoforms are functionally redundant and differ only by their spatio-temporal expression patterns. However, several studies in yeast and higher eukaryotic organisms challenged this widely accepted idea by demonstrating functional specificity among Hsp70 isoforms. Another element of complexity is brought about by specific cofactors, such as Hsp40s or nucleotide exchange factors that modulate the activity of Hsp70s and their binding to client proteins. Hence, a dynamic network of chaperone/co-chaperone interactions has evolved in each organism to efficiently take advantage of the multiple cellular roles Hsp70s can play. We summarize here our current knowledge of the functions and regulations of these molecular chaperones, and shed light on the known functional specificities among isoforms.

## INTRODUCTION

**Fig. (1):**
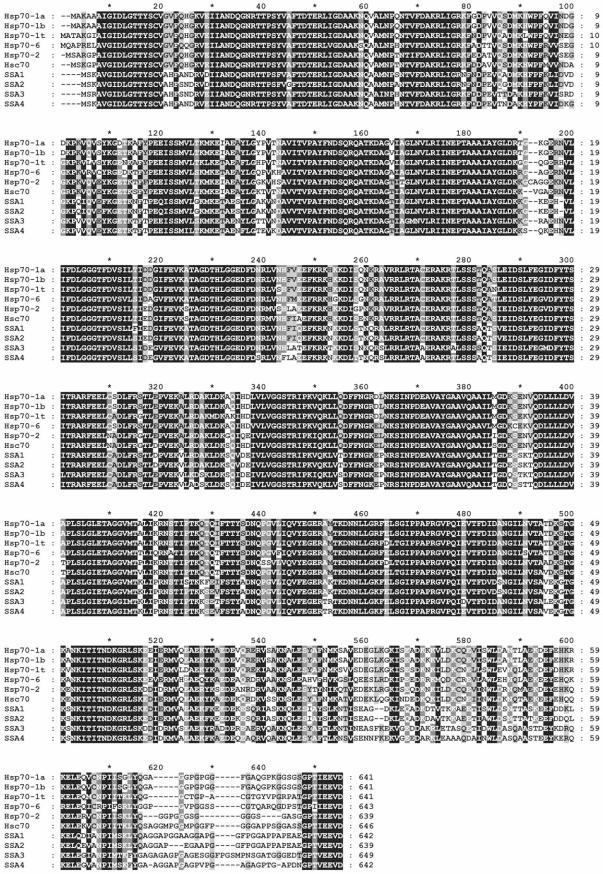
The alignment of yeast and human cytosolic Hsp70s shows their remarkable evolutionary conservation and highlights the variability of the C-terminal lid domain (this figure was made using clustalX 1.83 and GeneDoc v2.6).

The 70-kDa heat-shock proteins (Hsp70s) are a ubiquitous family of molecular chaperones found in all organisms and sub-cellular compartments where they play essential housekeeping functions in protein folding, synthesis, assembly, transport across biological membranes and degradation. They are also involved in quality control processes, such as protein refolding after a stress injury, and control the activity of regulatory proteins in signal transduction pathways [1]. Hsp70s constitute one of the most conserved protein families in evolution (Fig. **1**), and members of this family may be constitutively expressed or stress-inducible (hence their classification as heat-shock proteins) [2-6].

The functional pleiotropy of Hsp70s is achieved through the evolutionary amplification and diversification of *HSP70 *genes, cofactors that recruit and regulate Hsp70s for specific cellular functions, and cooperation of Hsp70s with other chaperone systems such as TRiC/CCT or Hsp90 [1]. All of these cellular activities depend on the ability of Hsp70s to interact with hydrophobic stretches of proteins in an ATP-dependent manner. Hsp70s are highly conserved and are composed of an N-terminal 44-kDa ATPase domain (also named adenine nucleotide-binding domain or NBD), an 18-kDa peptide-binding domain (PBD) and a C-terminal 10-kDa “lid” domain. The NBD has a bilobal structure and each globular lobe (I and II) is further conventionally divided into two subdomains (A and B) [7]. The interdomain linker connecting the NBD to the PBD is highly conserved and plays a critical role in the allosteric regulation of Hsp70s [8, 9]. Cytosolic Hsp70s also contain a G/P-rich C-terminal region containing an EEVD-motif that mediates their binding to Tetra-Trico-Peptide repeat (TPR)-domain containing co-chaperones such as the C-terminus-Hsp70-Interacting Protein CHIP. The EEVD motif is absent from specialized Hsp70s such as the ribosome-bound Ssb1/2p in yeast (see below).

Hsp70s cycle between two stable conformations with different affinities for substrates: in the ATP-bound state, Hsp70s display fast on-and-off rates of peptide binding, whereas in the ADP-bound state these constants are slowed, resulting in tighter association of the substrate within the PBD [10, 11]. The modulation of the affinity for polypeptide substrates is triggered by a conformational change in the lid that, upon ATP hydrolysis, closes on the substrate that is then trapped within the PBD [12]. It appears that ADP-ATP exchange is critical for substrate release and for the recycling of the molecular chaperone. This allosteric regulation of Hsp70s activity is tightly controlled by specific co-chaperones (Fig. **1A**).

**Fig. (2):**
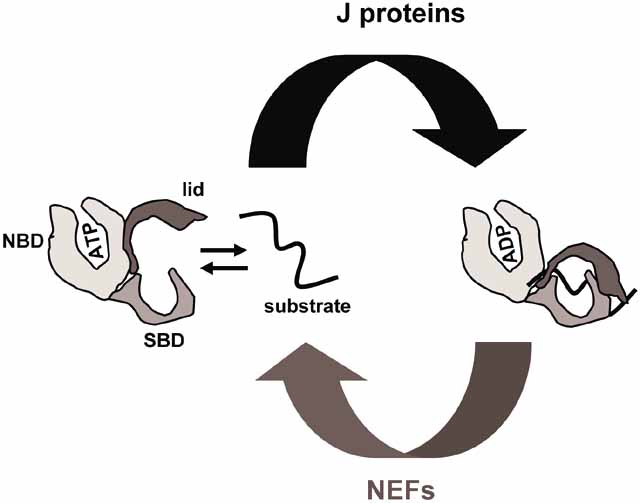
The Hsp70 ATPase cycle. In the ATP-bound state, Hsp70 have low affinity for substrates. Upon J-protein-stimulated ATP hydrolysis, a conformational switch of the lid tightly locks the substrate within the substrate binding domain. NEF catalyzed-ADP release and subsequent ATP re-binding trigger substrate release.

The well characterized Hsp40/DnaJ family members share a common ~70-amino-acid J-domain that mediates binding to Hsp70s and activation of their weak intrinsic ATPase activity (Fig. **2**). These cofactors, by virtue of their cellular localization and/or association to specific complexes, recruit Hsp70s to specific sites of the cell and therefore greatly contribute to the wide range of functions assigned to Hsp70s (reviewed in [13-17]).

Whereas ATP hydrolysis is stimulated by members of a common Hsp40/DnaJ family, ADP-ATP exchange is catalyzed by several evolutionary unrelated classes of nucleotide exchange factors (NEFs) (Fig. **2**); these include GrpE homologs in prokaryotes and the Bag1, HspBP1/Fes1p and Hsp110 families in eukaryotes (reviewed in [1, 18]). It should be noted that Hsp110s are molecular chaperones homologous in sequence and structure to Hsp70s and together these proteins form the super-family of Hsp70s [19, 20]. The domain architecture of Hsp110 is therefore very similar to that of Hsp70s; the main noticeable difference lies in large (~100-140 amino acids) acidic insertions between the PBD and the lid and also at the C-terminus [20]. Hsp110s are not protein folding chaperones, yet they act as efficient “holdases” by binding to and preventing aggregation of denatured proteins [21, 22]. Mammalian Hsp110 and yeast Sse1p were shown to functionally and physically interact with counterpart Hsp70s [23-25], and to be potent nucleotide-activated NEFs [26-28].

## THE EUKARYOTIC CYTOSOL CONTAINS MULTIPLE MEMBERS OF THE HSP70 SUPER-FAMILY

Genome sequencing efforts confirmed early observations that eukaryotes have several Hsp70 encoding genes [4]. Evidently, some of these Hsp70s are compartment specific and fulfil unique and essential functions in the endoplasmic reticulum, mitochondria or plastids. These organelle-specific Hsp70s are generally encoded by a single gene in most organisms. Strikingly, multiple Hsp70 isoforms very often coexist in the same cytosol and are encoded by different homologous genes. In Table (**1**) we describe the cytosolic Hsp70 machinery of various fully sequenced eukaryotic organisms, ranging from yeast to human. For most of these selected species, the Hsp70 system has been recently described in details at the genomic and/or functional levels [6, 29-34].

With the exception of ribosome-associated specialized Hsp70s (that do not contain the C-terminal EEVD motif), canonical Hsp70s are generally believed to be functionally redundant, the main differences between isoforms lying in their spatio-temporal expression. This is exemplified by studies in yeast that showed that only one member of the Ssa group of Hsp70s can support yeast growth if expressed at sufficient levels [29]. Additionally, heterologously expressed Hsp70s were shown to protect mammalian cells and transgenic animals from various stresses, demonstrating that the protective role of these molecular chaperones is highly conserved [35-38].

However, many studies also raised the possibility of functional specificity among Hsp70 isoforms, meaning that a particular Hsp70 will for instance preferentially bind to a subset of client proteins and/or co-chaperones to perform a unique task. Such a specialization among Hsp70s may explain the need for several Hsp70-encoding genes and their different tissue-expression patterns in complex multicellular organisms. In this review, we will discuss examples of functional specificities among Hsp70s in yeast and humans, hypothesize on the molecular basis that may govern this specificity, and suggest directions for future research in this field.

## REDUNDANT AND SPECIALIZED FUNCTIONS AMONG HSP70S IN YEAST

### The Cytosolic Hsp70 System in Yeast

The yeast *Saccharomyces cerevisiae* contains seven cytosolic Hsp70s that fall into two major groups: 1) the canonical Ssa1, Ssa2p, Ssa3p and Ssa4p proteins and 2) the ribosome-associated Ssb1p, Ssb2p and Ssz1p proteins (Table **1**) [4].

The highly homologous Ssa proteins differ by their expression pattern: the *SSA2 *gene is constitutively expressed at high levels; the *SSA1 *gene is constitutively expressed, yet at lower levels than *SSA2,* and is also induced by stress; the *SSA3 *and *SSA4 *genes are not expressed during normal vegetative growth, but their expression is dramatically induced upon stress; additionally, Ssa3p levels are increased upon entry into stationary phase [29, 39-42]. The simultaneous deletion of these four genes is lethal, but can be complemented by the over-expression of either one of them, suggesting redundant functions for these molecular chaperones [29]. It should be noted that a Δ*ssa1*Δ*ssa2* mutant is viable and constitutively thermotolerant because of the upregulation of *SSA3*, *SSA4* and other components of the general stress response [29, 39, 40]*. *However, the Δ*ssa1*Δ*ssa2* mutant forms small colonies at 23°C and is thermosensitive for growth at 37°C, suggesting that the functions carried by Ssa1p and/or Ssa2p cannot be fully complemented by the induction of *SSA3 *and *SSA4* [29, 39, 40].

The nearly identical Ssb1p and Ssb2p proteins interact with nascent chains at the yeast ribosome and provide a dispensable function in the process of translation and in early folding events [43, 44]. These Hsp70s are unable to complement the lack of Ssa proteins, indicating that these two groups of Hsp70s clearly have distinct non overlapping functions [4]. Ssb1p and Ssb2p are recruited to the ribosome through their interaction with the stable heterodimeric Ribosome-Associated Complex (RAC) that is composed of the J-domain protein Zuo1p (or zuotin) and the atypical Hsp70 Ssz1p [45, 46]. The simultaneous deletion of *SSB1* and *SSB2 *results in slow growth, cold sensitivity and hypersensitivity to translation-inhibiting drugs such as hygromycin B or paromomycin. Identical phenotypes are observed upon deletion of *SSZ1* or *ZUO1*, suggesting that Ssb1/2p and RAC form a functional triad *in vivo *[45-48]. The Ssb1/2p proteins are not stimulated by other J-proteins than Zuo1p and require their PBD for function [49, 50]. The Ssz1p protein diverges markedly from other Hsp70s: it contains an unusually short PBD and lacks the C-terminal lid domain; in fact, truncated Ssz1p lacking nearly the entire PBD is fully functional *in vivo *[51]; Ssz1p is not an ATPase *in vitro* and its nucleotide binding ability is not strictly required *in vivo* [52]. Ssz1p has been proposed to serve as a structural scaffold between Ssb1/2p and Zuo1p [53], although this may not be its only function [52]. While Ssb proteins are generally considered fungal-specific, we have identified an uncharacterized Ssb homolog in *C. elegans* (Table **1**). Moreover, a mammalian RAC complex has been recently described, and is comprised of the Zuo1 homolog MPP11 and the Ssz1 homolog Hsp70L1 (or Hsp70-14; Table **1**) [54].

In *S. cerevisiae*, the Ssa proteins are recruited and activated by 11 cytosolic J-proteins [55], and both Ssa and Ssb proteins can be regulated by the Fes1p and Sse1/2p NEFs [23, 24, 26, 27, 56, 57]. Whether the different Ssa isoforms have preferences and/or different affinities for distinct J-proteins or NEFs has not been systematically tested, but remains an attractive possibility that could partly explain the functional specificities observed among Hsp70 isoforms. Indeed, a functional distinction between cytosolic, ER or mitochondrial Hsp70s has been greatly documented and was attributed to specific interactions between these Hsp70s and their partner J-proteins. In particular, cytosolic and ER lumenal Hsp70s could not replace one another in *in vitro* reconstitution assays of protein translocation across the ER membrane [58, 59]. Moreover, mitochondrial Hsp70 was unable to replace the ER lumenal Hsp70 in similar assays [60]. In both cases, this has been attributed to impaired interactions of the “extraneous” Hsp70 with the “endogenous” compartment-specific J-proteins, suggesting convergent evolution of chaperone and co-chaperones for proper interaction [16, 58, 60].

As shown in Table (**1**), Ssa, Ssb and Ssz homologues are present in most yeasts that mainly differ with respect to the number of Ssa-encoding genes: only two in *Schizosaccharomyces pombe* and *Candida albicans*, and four in *Y. lipolytica*. We are currently investigating the regulations and functions of the four *Y. lipolytica *Ssa proteins that we have named Ssa5-8 to avoid confusion with the *S. cerevisiae *Hsp70 system (Martineau C.N. and Kabani M., unpublished data). A comparison of the Hsp70 systems in these two distantly related yeasts will provide useful information on the plasticity and dynamics of Hsp70-containing chaperone networks.

### Examples of Functional Specificity Among the Ssa Group of Hsp70s

Early evidence for functional specificity among Ssa proteins was brought by a study from Eisenberg and collaborators that investigated the ability of yeast cytoplasmic Hsp70s to dissociate clathrin from bovine brain coated vesicles [61]. Using purified Hsp70 preparations from wild-type yeast, they first showed that the *in vitro *clathrin uncoating activity is associated with Ssa but not Ssb proteins. They then showed that higher uncoating activities could be achieved using Hsp70 preparations from a Δ*ssa1* mutant than from a Δ*ssa2* mutant. Because Ssa3p and Ssa4p are nearly undetectable in these preparations, they concluded that Ssa2p has a markedly higher uncoating activity than Ssa1p. Moreover, they showed that Ssa1p inhibits the uncoating activity of Ssa2p, probably by blocking Hsp70-binding sites on clathrin baskets [61]. The molecular basis of this functional difference in clathrin uncoating between these highly homologous Hsp70s remains to be directly addressed. One possible explanation is that Ssa2p and Ssa1p could have markedly different affinities for clathrin and/or the J-protein auxilin (Swa2p) which is specifically required to activate Hsc70 during endocytosis [62]. In humans and fruit fly, clathrin uncoating was similarly shown to depend on Hsc70 and Hsc70-4, respectively, which are the most abundant and constitutive Hsp70s in these organisms [63, 64]. This is not surprising given that Hsp70 acts stoichiometrically in clathrin uncoating, and therefore fair amounts of the molecular chaperone are required to fulfil this vital cellular process [65].

Another functional distinction between Ssa1p and Ssa2p has been reported with respect to their roles in prion propagation. The yeast epigenetic factors [PSI+], [URE3] and [PIN+] are the prion forms of the Sup35p, Ure2p and Rnq1p proteins, respectively [66-68]. These infectious proteins self-propagate as amyloids [66, 67, 69-72] and therefore constitute a powerful and safe model to investigate mammalian prion-related diseases such as sheep scrapie or transmissible spongiform encephalopathies [73]. Molecular chaperones of the Hsp104, Hsp70 and Hsp40 families play critical roles in prion formation and propagation [74, 75]. The fungal specific Hsp104 molecular chaperone promotes the ATP-dependent solubilization of aggregated misfolded proteins with the help of Hsp70 and Hsp40 [76]. Hsp104 is strictly required for yeast prion propagation, as the deletion of the *HSP104* gene or the inactivation of Hsp104 by growth in the presence of 5 mM guanidium chloride cure cells from [PSI+], [URE3] and [PIN+]. In contrast, the overexpression of Hsp104 cures [PSI+] but not [URE3] [67, 77-80]. Hsp104 was proposed to mediate protein-only inheritance by remodelling large prion aggregates into new self-replicating particles (or seeds) [69, 81-83]. The effects of Hsp70s and Hsp40s on prion propagation are complex and prion-dependent [75]. Mutation of the cytosolic Hsp70 Ssa1p impairs [PSI+] propagation while its overexpression was shown to inhibit [PSI+] curing by overexpressed Hsp104 [84, 85]. The overproduction of Ssa1p, but not Ssa2p, cures [URE3] [86], whereas mutations in *SSA2,* but not *SSA1,* impair [URE3] propagation [87]. The overexpression of Ydj1p, the most abundant yeast Hsp40, cures cells from [URE3] [78], whereas mutations in Sis1p cure [PIN+] [88]. Similarly, NEFs have been shown to influence prion propagation. Daniel Masison and collaborators have isolated an *SSA1-21* mutation that weakens [PSI+] propagation [84]. They then showed that in the *SSA1-21 *background the depletion of Fes1p further destabilized [PSI+], whereas its overexpression restored almost normal [PSI+] propagation [89]. However, contrasting results have been reported for [PSI+] propagation when *FES1* is deleted in an otherwise wild-type background [89, 90]. The deletion of *SSE1 *and *FES1 *completely blocked [URE3] propagation, but only the overexpression of Sse1p cured it [90-92]. In addition, the deletion of *SSE1* severely inhibited [PSI+] formation while the overexpression of Sse1p improved it [92]. The Hsp70 machinery appears as an important modulator of amyloid formation and protein-only inheritance, and the complex phenotypes obtained upon mutations in its components indicates that a dynamic network of specific chaperone/co-chaperone/substrate interactions occur *in vivo*. A better understanding of the role of the Hsp70 system in the process of amyloidogenesis will benefit from *in vitro* approaches where the effects of purified chaperones and co-chaperones on the assembly of amyloid fibrils can be precisely tested [93, 94].

We have recently showed that mutations in components of the cytosolic Hsp70 machinery (*e.g. FES1, SSE1, YDJ1, SSA1) *dramatically impair biofilm (or ‘mat’) formation in yeast [95]. We showed that the overexpression of *FES1* rescues the defect in biofilm formation of a Δ*sse1* strain, but the opposite is not true: the Δ*fes1* mutant is unable to form ‘mats’ on semi-solid medium with or without *SSE1/2* overexpression [95]. Thus, Fes1p and Sse1/2p play both overlapping and distinct functions as suggested by earlier studies [27, 96]. Remarkably, we showed that a Δ*ssa1* mutant, in an otherwise wild-type background for the other SSA genes, is severely affected in biofilm formation [95]. The deletion of *SSA2* had more subtle effects suggesting that Ssa1p is specifically required for ‘mat’ formation [95]. Moreover, we showed that the additional deletion of *SSA3* and *SSA4* aggravated the phenotypes of the Δ*ssa1* and Δ*ssa2* mutants, indicating a possible functional cooperation between the constitutive and inducible Hsp70s [95].

## THE HUMAN HSP70 SYTEM

### The Cytosolic Hsp70 System in Humans

Several reviews and studies attempted to provide an accurate description of the human Hsp70 family, a task that has been complicated by confusing and conflicting nomenclature in the literature [6, 34, 97]. However, extensive literature and database search, facilitated by the availability of the human genome, recently allowed Alberto Macario and collaborators to provide a detailed description of human Hsp70 encoding genes [34]. They identified up to 17 Hsp70-encoding genes that fall into evolutionary and functionally distinct groups, as well as 30 pseudogenes [34]. For each of these genes, an impressively high number of mRNA variants and isoforms were identified through EST analysis, although their functional relevance is still unknown [34]. In Table (**1**), we chose to present only the *bona fide* human cytosolic Hsp70s for which expression has been proved and that correspond to Ssa or Sse-like proteins [6, 34, 97].

The human cytosolic Hsp70s are comprised of six canonical members, named Hsp70-1a, Hsp70-1b, Hsp70-1t, Hsp70-2, Hsp70-6 and Hsc70, and of an Ssz-like protein named Hsp70-14 (or Hsp70L1) [6, 34, 97] (Table **1**). These proteins were also described with different acronyms that are indicated in Table (**1**). While the expression of Hsp70-1a, Hsp70-1b and Hsp70-6 is strongly induced by stress [6, 34], all these cytosolic Hsp70s differ considerably with respect to their expression patterns in the different tissues and developmental stages, as predicted by assessing the relative number of ESTs per tissue type [34].

The heat-shock cognate Hsc70 is by far the most expressed Hsp70 in all tissues with particularly high levels in the vascular tissue [34], and is believed to play essential house-keeping functions in protein folding, transport across biological membranes, prevention of protein aggregation and uncoating of clathrin vesicles [6, 98]. The intron-less genes encoding Hsp70-1A, Hsp70-1B and Hsp70-1t are all closely linked and located within the same MHC class III region on chromosome 6 [99, 100], yet their expression patterns differ [6, 34, 101]. Hsp70-1a and Hsp70-1b are expressed in most tissues, and in most cases Hsp70-1a levels are much higher than Hsp70-1b levels [6, 34]. Moreover, very high expression levels were observed for Hsp70-1a in the spleen [34]. On the other hand, Hsp70-1t is expressed at almost undetectable levels in most tissues, except in testis, adipose tissue and pituitary glands where its levels are much higher [6, 34, 101]. In contrast to Hsp70-1a and Hsp70-1b, the levels of Hsp70-1t are not induced by heat stress [101]. Hsp70-2 is expressed at low to undetectable levels in most tissues, and at high levels in testis, brain and umbilical cord [6, 34]. The expression of Hsp70-6 is only induced after severe stress insults [102, 103], although moderate levels of this protein have been reported in some blood cells as well as in the bladder, trachea and esophagus [6, 34].

Thus, all cytosolic Hsp70s are constitutively expressed at varying levels making each tissue and developmental stage unique in its Hsp70 content [6, 34]. Important efforts will be needed to define the specific roles of each Hsp70 in each cell and tissue type, taking into account the possibility that these Hsp70s may functionally interact with one another, either positively or negatively, depending on their respective abundance. Whether the Hsp70 co-chaperones content also varies among cell and tissue types has yet to be determined, but could also be a major determinant of the functional specialization of Hsp70s.

### Examples of Functional Specificity Among the Human Cytosolic Hsp70s

Understanding the need for up to six Hsp70 family members in human cells and sorting out their redundant from their specialized functions have proven difficult and technically challenging. Most of the published data on human Hsp70s were obtained by studying the constitutive Hsc70 or the major heat-inducible Hsp70 (which in fact refers to both Hsp70-1a and Hsp70-1b) that were often considered as equivalent and functionally interchangeable. [6, 104]. However, a clear distinction between constitutive and inducible Hsp70 isoforms emerged from several studies, including the characterization of mouse knock-out models [6]. It appeared very logically that constitutive Hsp70s mostly play important housekeeping functions while inducible Hsp70s are required to cope with stress situations [6].

Hence, Hsc70 was shown to be essential for viability in vertebrates, including fruit fly [105], mouse [106] and human cell lines [107, 108], whereas mice deficient for the homologues of human Hsp70-1a and Hsp70-1b (Hsp70.1 and Hsp70.3) develop normally in standard conditions but show significantly decreased resistance to stress situations such as heat shock, cardiac ischemia or radiation [6, 109-117].

Additionally, Hsp70 and Hsc70 were shown to have differential and antagonistic effects with regard to the intracellular trafficking of ENaC, an epithelial sodium chloride channel: Hsp70 promoted the maturation and functional cell surface expression of ENaC in *Xenopus* oocytes, whereas Hsc70 disfavoured it [104]. Similarly, both the overexpression of Hsp70 and the decrease of Hsc70 levels *via *the use of sodium 4-phenylbutyrate were shown to promote the intracellular trafficking of the ΔF508 mutant of the cystic fibrosis transmembrane conductance regulator (CFTR) [118-120].

Hsp70 levels are abnormally high in a wide variety of tumor cell types and contribute to tumorigenesis and resistance to chemotherapy by directly interfering with several key components of the apoptotic signalling pathway [121]. Similarly, an up-regulation of the HspBP1 NEF was observed in various tumor cell types [122, 123]. HspBP1 has been shown to modulate Hsp70s activity either negatively [124, 125] (at high HspBP1/Hsp70 molar ratios) or positively (at low HspBP1/Hsp70 molar ratios) [126]. Interestingly, HspBP1 was shown *in vivo* to bind with a much higher affinity to the stress-inducible Hsp70 than to the abundant and constitutively expressed Hsc70. Hence, HspBP1 is present in great excess (~10 fold) compared to its preferential Hsp70 partner in normal cells. However, the HspBP1/Hsp70 molar ratio may be lowered considerably in tumor cells (from ~10 to ~2) because the up-regulation of Hsp70 is greater than that of HspBP1 in these cells [123]. This may contribute to the Hsp70-mediated resistance to chemotherapy, as tumor cells with high HspBP1/Hsp70 molar ratios were much more susceptible to anticancer drugs than were those with a low ratio [123]. In addition, anticancer drugs up-regulated HspBP1 in various cancer cells, while no effect was observed on Hsp70 levels [123]. These findings suggest that HspBP1 antagonizes the prosurvival function of Hsp70 [123]. In support of this hypothesis, RNAi-mediated depletion of HspBP1 markedly reduced the susceptibility of tumor cells to anticancer drugs [123]. Conversely, transient ectopic expression of HspBP1 in tumor cells enhanced the cathepsin-mediated cell death induced by anticancer drugs [123]. This pro-apoptotic function of HspBP1 appeared to be dependent on its ability to bind to Hsp70 and was inhibited by heat-shock induced up-regulation of Hsp70 [123]. These findings exemplify that part of the functional distinction among Hsp70 isoforms may lie in differential interaction with their co-chaperones, thereby forming specific functional networks in each cell or tissue type. Remarkably, both primate and plant Hsc70 were able to support growth of a Δ*ssa1-4* yeast mutant, whereas the corresponding inducible Hsp70s did not [127], suggesting an evolutionary conserved functional distinction among these molecular chaperones.

The Hsp70-2 protein appears to play an important role in spermatogenesis in humans and mice *via *its essential chaperoning functions for the cyclinB/cdc2 complex during meiosis, and for spermatid DNA-packaging proteins involved in post-meiotic genome reorganization [6, 128-132]. Additionally, Hsp70-2 is upregulated in some primary and metastatic breast cancers, and was shown to be required for the growth and survival of several human cancer cells [6, 107, 108, 133]. The depletion of Hsp70 or Hsp70-2 from cancer cells resulted in markedly different phenotypes and gene expression profiles, whereas their concomitant depletion resulted in synergistic antiproliferative effects, suggesting separate non-overlapping functions for these Hsp70s [108].

By virtue of their cellular protective role, Hsp70s are important candidates in gene-longevity association studies [134, 135]. Indeed, the ability to cope with cellular stress by the induction of Hsp70 was shown to decline with age in numerous *in vivo *and *in vitro* models [134, 136-139]. Moreover, the presence of extra-copies of a heat-inducible Hsp70-encoding gene increased the lifespan of transgenic fly models after a transient mild heat-shock [140]. Specifically, polymorphisms positively or negatively associated with longevity were identified in the genes encoding the MHC-III-linked Hsp70-1a, Hsp70-1b and Hsp70-1t proteins, suggesting that unique anti-ageing functions are fulfilled by these isoforms (reviewed in [134, 135]).

## CONCLUSIONS AND FUTURE DIRECTIONS: MOLECULAR BASIS OF HSP70 SPECIALIZATION

Despite a high degree of sequence conservation and overlapping chaperoning functions, members of the Hsp70 superfamily appear to have evolved specialized functions for which they can not replace each other. This functional specificity is most apparent and widely accepted for compartment-specific and ribosome-associated Hsp70s, and also for the Hsp110 family members that evolved to act as Hsp70 NEFs [6, 98]. However, less attention has been paid to the possibility that a similar specialization might occur among the highly conserved cytosolic Hsp70s, although it may provide an answer to why some organisms such as humans or *Drosophila melanogaster* need up to 6 or 10 cytosolic Hsp70s, respectively (Table **1**). A recent study demonstrated functional differences among highly conserved ribosomal protein paralogs in yeast, indicating that the co-existence of multiple members of a given protein family is not only the result of gene duplication and mere redundancy, but also significantly contributes to cellular homeostasis [141].

Future efforts will have to identify and characterize the unique functions played by individual Hsp70s in particular cell or tissue types. Several non-mutually exclusive hypotheses can be made, according to our current knowledge of Hsp70 function, to understand the molecular basis of such specialization. A first model would be that Hsp70 isoforms differentially interact with the various cytosolic Hsp70 co-chaperones, as observed for the preferred interaction of HspBP1 with Hsp70 and not with Hsc70 [123]. A second model would be that Hsp70 isoforms bind to different sets of client proteins or to different types of substrates (*e. g. *native client proteins, nascent proteins, misfolded proteins, protein aggregates, etc.). In support of this hypothesis, different peptide binding specificities were described for bacterial DnaK, mammalian Hsc70 and BiP [142, 143]. Similarly, the DnaK protein from the archea *Methanosarcina mazei *failed to fully complement a *dnak *mutant in *Escherichia coli*, and was shown to have markedly different peptide binding properties [144, 145]. Importantly, the 10-kDa C-terminal domain of Hsp70s is the less conserved region and could play an important role in modulating Hsp70-substrate interactions (Fig. **1**). Additionally, different Hsp70 isoforms could cooperate in the process of protein folding by binding to different exposed sites on a given substrate. To our knowledge, the side-by-side and thorough biochemical characterization of Hsp70 isoforms from a single model organism has not been reported but would certainly help clarifying the functions and regulations of these essential folding enzymes. Because Hsp70s are relevant targets for drug-based treatments for cancers or protein folding disorders, the identification of the molecular determinants that govern their functional specificities in particular cell or tissue types will greatly improve the efficacy of these approaches without unwillingly affecting vital cellular processes.

## Figures and Tables

**Table 1.** The Cytosolic Hsp70s of Various Eukaryotes are Shown Together with Some Important Co-Chaperones (Because of their High Number, J-Proteins are not Shown). UniProt Accession Numbers are Provided in Most Cases, Along with the Alternative Acronyms by which these Proteins were Described when Appropriate

	Yeasts				Nematode	Green Algae	Ascidian	Plant	Fruit fly	Human
	*S. cerevisiae*	*S. pombe*	*C. albicans*	*Y. lipolytica*	*C. elegans*	*C. reinhardtii*	*C. intestinalis*	*A. thaliana*	*D. melanogaster*	*H. sapiens*
Canonical Hsp70's	Ssa1p (P10591)	*Sp.*Ssa1p (Q10265)	*Ca.*Ssa1p (P41797)	Ssa5p (Q6C0E9)	Hsp70-1 (P09446)	*Cr.*Hsp70-3 (P25840)	*Ci.*HSPA1/6/7-like (P91874)	*At.*Hsp70-1 (P22953)	Hsp70Aa (P82910)	Hsp70-1a (Hsp70, Hsp72, Hsp70-1) (P08107)
	Ssa2p (P10592)	*Sp.*Ssa2p (O59855)	*Ca.*Ssa2p (P46587)	Ssa6p (Q6C3G5)	Hsp70-7 (Q9XTL8)		*Ci.*HSPA2/8	*At.*Hsp70-2 (P22954)	Hsp70Ab (P02825)	Hsp70-1b (Hsp70, Hsp72, Hsp70-1) (P08107)
	Ssa3p (P09435)			Ssa7p (Q6C9V0)	Hsp70-8 (Q9XTL8)			*At.*Hsp70-3 (O65719)	Hsp70Ba (Q8INI8)	Hsp70-1t (Hsp70-hom) (P34931)
	Ssa4p (P22202)			Ssa8p (Q6C864)	Hsp70-9 (O45246)			*At.*Hsp70-4 (Q9LHA8)	Hsp70Bbb (Q9VG58)	Hsp70-2 (Hsp70-3) (P54652)
								*At.*Hsp70-5 (Q959N1)	Hsp70Bb (Q9BIS2)	Hsp70-6 (Hsp70B') (P17066)
									Hsp70Bc (Q9BIR7)	Hsc70 (Hsp70-8, Hsp73) (P11142)
									Hsc70-1 (P29843)	
									Hsc70-2 (P11146)	
									Hsc70-4 (P11147)	
									Hsp68 (097125)	
Other Hsp70's	Ssb1p (P11484)	*Sp.*Ssb1p (Sks2) (Q10284)	*Ca.*Ssb1p (P87222)	*Yl.*Ssb1p (Q6CIA7)	Hsp70-10 (Q9TW52)	-	-	-	-	-
	Ssb2p (P40150)									
	Ssz1p (P38788)	*Sp.*Ssz1p(P87142)	*Ca.*Ssz1p (Q5A678)	*Yl.*Ssz1p (Q6CEW0)	-	-	-	-	-	Hsp70-14 (Hsp70L1) (Q0VDF9)
Hsp110's	Sse1p (P32589)	*Sp.*Sse1p (Pss1) (O59838)	*Ca.*Sse1p (Q96VB9)	*Yl.*Sse1p (Q6C618)	Hsp110-1 (Q05036)	-	*Ci.*HSPA4/4L/HSPH1	*At.*Hsp70-14 (Q957C0)	Hsc70Cb (Q9XZT5)	Hsp110 (Hsp70-4) (P34932)
	Sse2p (P32590)							*At.*Hsp70-15 (Q9CA95)		Hsp110 (Hsp70-4L) (O95757)
								*At.*Hsp70-16 (A8MRM9)		Hsp105 (Q92598)
HspBP1	Fes1p (P38260)	*Sp.*Fes1p (O43030)	*Ca.*Fes1p (Q59NN8)	*Yl.*Fes1p (Q6C239)	-	-	-	*At.*HspBP1-1 (Q9M346)	?	HspBP1 (Q9NZL4)
								*At.*HspBP1-2 (Q84J81)		
								*At.*HspBP1-3 (Q9LZL7)		
Bag proteins	Snl1p (P40548)	Bag-1A(O59739)	*Ca.*Snl1p (Q59NB3)	*Yl.*Bag1 (Q6C9J3)	*Ce.*Bag-1 (044739)	-	*CI.*BAG1	*At.*BaBag-1 (Q0WUQ1)	starvin, isoform A (Q9VU81)	Bag-1L, Bag-1M, Bag-1S (Q99933)
		Bag-1B (O59739)			Unc-23a (O61980)		*CI.*BAG3	*At.*Bag-2 (Q8LEP8)	starvin, isoform B (Q9VU83)	Bag-2 (O95816)
					Unc-23b (Q86S24)		*Ci.*BAT3	*At.*Bag-3 (Q9LYP4)	starvin, isoform C (Q9VU82)	Bag-3 (O95817)
					Unc-23c (Q5TKA9)			*At.*Bag-4 (O65021)		Bag-4 (O95429)
								*At.*Bag-5 (O65373)		Bag-5 (Q9UL15)
								*At.*Bag-6 (O82345)		Bag-6, BAT3 (P46379)
								*At.*Bag-7 (Q9LVA0)		
CHIP	-	-	-	*Yl.*Chn1p (Q6C1F4)	Chn-1 (Q9BMU2)	*Cr.*CHIP (A8J756)	-	*At.*CHIP (Q9SRS9)	*Dm.*CHIP (Q9XYW6)	CHIP (Q9UNE7)
